# The Self-help Online against Suicidal thoughts (SOS) trial: study protocol for a randomized controlled trial

**DOI:** 10.1186/s13063-017-1794-x

**Published:** 2017-01-28

**Authors:** Charlotte Mühlmann, Trine Madsen, Carsten Hjorthøj, Ad Kerkhof, Merete Nordentoft, Annette Erlangsen

**Affiliations:** 1 Danish Research Institute for Suicide Prevention, Mental Health Centre Copenhagen, Kildegårdsvej 28, 2900 Hellerup, Denmark; 2 Copenhagen University Hospital, Mental Health Centre Copenhagen, Kildegårdsvej 28, 2900 Hellerup, Denmark; 3 Department of Clinical, Neuro, and Developmental Psychology and the EMGO Institute for Health and Care Research, Faculty of Behavioural and Movement Sciences Vrije Universiteit Amsterdam, Van der Boechorststraat 1, 1081 BT Amsterdam, The Netherlands; 4 Department of Mental Health, John Hopkins Bloomberg School of Public Health, Baltimore, MD USA; 5 Department of Regional Health Research, University of Southern Denmark, Odense, Denmark

**Keywords:** Suicidal thoughts, Suicidal ideation, Randomized controlled trial, Internet intervention, Online intervention, Cognitive behavioral therapy, Self-help

## Abstract

**Background:**

Suicidal thoughts are common, causing distress for millions of people all over the world. However, people with suicidal thoughts might not access support due to financial restraints, stigma or a lack of available treatment offers. Self-help programs provided online could overcome these barriers, and previous efforts show promising results in terms of reducing suicidal thoughts. This study aims to examine the effectiveness of an online self-help intervention in reducing suicidal thoughts among people at risk of suicide.

The Danish Self-help Online against Suicidal thoughts (SOS) trial is a partial replication of a previously conducted Dutch trial.

**Methods and design:**

A randomized, waiting-list controlled trial with 1:1 allocation ratio will be carried out. A total of 438 people with suicidal thoughts will be recruited from the Danish suicide hotline, The Lifeline’s, website and allocated to the intervention condition (*N* = 219) or the control condition (*N* = 219).

The intervention condition consists of a 6-week, Internet-based self-help therapy intervention. The format of the intervention is self-help, but the participants can be guided by the trial manager. The control condition consists of a waiting-list assignment for 32 weeks.

The primary outcomes are frequency and intensity of suicidal thoughts. Secondary outcome measures include depressive symptoms, hopelessness, worrying, quality of life, costs related to health care utilization and production loss. Number of deliberate self-harm episodes, suicides and deaths will, as well as the participant’s evaluation of the intervention and the experience of negative effects, be investigated. Assessments will be conducted over the intervention website through self-report questionnaires at baseline, 2 weeks, 4 weeks, 6 weeks and 32 weeks (6 months post intervention).

**Discussion:**

If we find the intervention to be linked to reductions in suicidal thoughts, this will strengthen the evidence that online self-help interventions are relevant tools for people with suicidal thoughts.

**Trial registration:**

ClinicalTrials.gov, NCT02872610. Registered on 9 August 2016.

**Electronic supplementary material:**

The online version of this article (doi:10.1186/s13063-017-1794-x) contains supplementary material, which is available to authorized users.

## Background

The World Health Organization estimates that suicide accounts for over 800,000 deaths each year [1] and the lifetime prevalence of suicidal ideation is found to be 9.2%; i.e., in one in ten persons [2].

Different support options exist for people with suicidal thoughts. In Denmark, people at risk of suicide can receive free specialized psychosocial therapy in Suicide Prevention Clinics [3]. Some of these clinics have been operating since 1992, and the treatment seems to be effective [3]. Another support option is The Lifeline which provides free and anonymous support online and via telephone [4]. However, even in a high-income country like Denmark with various support options, the relatively stable suicide rate over the last 10 years indicates that not all people with suicidal thoughts seek or benefit from the existing support options. Internet-based therapy is still at a very early stage in Denmark. In 2016 only a couple of studies in Denmark were investigating the effect of the few Internet-based therapy programs that exist, but findings from other countries indicate that clinically and cost-effective online treatment options exist for depression and anxiety disorders [5, 6]. A Dutch randomized controlled trial found that an online self-help intervention for people with suicidal thoughts was effective in lowering suicidal thoughts and cost-effective [7, 8].

The objective of the Danish Self-help Online against Suicidal thoughts (SOS) trial is to examine whether an Internet-based self-help intervention, provided to people with suicidal thoughts, is superior to a waiting-list control condition in reducing suicidal thoughts. Key secondary outcomes of this study cover psychological wellbeing as well as costs related to health care utilization and production loss.

The study is a partial replication of the aforementioned Dutch trial [7]. While the previous trial excluded people with severe suicidal thoughts and did not extend the follow-up period to post treatment, the Danish trial aims to address these issues.

## Methods and design

### Design

The SOS trial is designed as a randomized, controlled trial with a waiting-list control and with a 1:1 allocation ratio.

The primary hypothesis is that the intervention is superior to the control condition in reducing suicidal thoughts using the Beck Scale for Suicide Ideation at post test (6 weeks). Our secondary hypothesis is that the effect will be maintained at 6-month follow-up and that the program will be effective in improving the participant’s psychological wellbeing at post test and at follow-up. Table 1 shows the entries to the World Health Organization Trial Registration Data Set.

**Table 1:** World Health Organization Trial Registration Data Set

Data category	Information
Primary registry and trial identifying number	ClinicalTrials.gov NCT02872610.
Date of registration in primary registry	18 August 2016
Secondary identifying number	TrygFonden 106690
	The Research Ethics Committee of the Capital Region of Denmark H-15002490
Source(s) of monetary or material support	TrygFonden reference number 106690
Primary sponsor	Merete Nordentoft, Mental Health Centre Copenhagen
Contact for public queries	CM: Charlotte.Muehlmann@regionh.dk
	MN: Merete.nordnetoft@regionh.dk
	AE: Annette.erlangsen@regionh.dk
Contact for scientific queries	CM, MN, AE Charlotte.Muehlmann@regionh.dk
	Merete.nordnetoft@regionh.dk
	Annette.erlangsen@regionh.dk
	Mental Health Centre Copenhagen
Public title	The Self-help Online against Suicidal thoughts (SOS) trial – a Danish randomized controlled waiting-list trial for people with suicidal thoughts
Scientific title	The Self-help Online against Suicidal thoughts (SOS) trial – a Danish randomized controlled waiting-list trial for people with suicidal thoughts
Countries of recruitment	Denmark
Health condition(s) or problem(s) studied	Suicidal thoughts
Intevention(s)	An Internet-based self-help intervention for people with suicidal thoughts
	Waiting-list control condition
Key inclusion and exclusion criteria	Ages eligible for study: ≥18 years; sexes eligible for study: both
	Inclusion criteria: 18 years or older; sufficient command of the Danish language; have a personal code card (NemID)
	Exclusion criteria: no suicidal thoughts (defined as a cutoff score of <3 on the Beck Scale for Suicide Ideation)
Study type	Interventional
	Allocation: randomized
	Intervention model: parallel assignment
	Primary purpose: to lower the participant’s degree of suicidal thoughts
Date of first enrolment	August 2016
Target sample size	438
Recruitment status	Pending
Primary outcome(s)	Outcome: suicidal thoughts
	Method of measurement: Beck Scale for Suicide Ideation
	Time point: 2, 4 and 6 weeks after baseline, with time × group interaction test as indicator of efficacy
Key secondary outcomes	Suicidal thoughts, depression, hopelessness, worrying, quality of life, health care utilization, medication prescriptions, production loss, episodes of deliberate self-harm, death by suicide, negative effects of the intervention and evaluation and utility of the self-help program

### Sample and recruitment

**Fig. 1:**
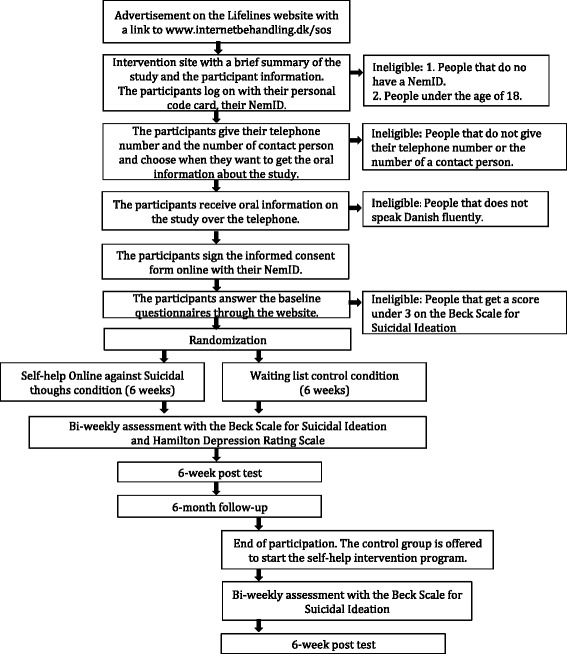
Flow diagram for the Self-help Online against Suicidal thoughts (SOS) trial

The target population is adults in Denmark with suicidal thoughts. The study will be announced on The Lifeline’s website, and their volunteers will inform users about the study and refer to the web page listing where the study is described. Psychiatric hospitals and other outpatient clinics of relevance in the Capital Region of Denmark will be provided with information on the project, and are encouraged to refer patients with suicidal thoughts to The Lifelines website. See Fig. 1 for the trial flow diagram.

### Eligibility criteria

To be eligible for enrollment, participants will have to: (1) be 18 years or older, (2) have a NemID, a personal code card used in Denmark for digital communication with authorities and (3) be fluent in Danish. Participants will be excluded from the study if they (1) do not provide their own telephone number and the telephone number of a contact person or (2) if no suicidal thoughts are noted (defined as a cutoff score of <3 on the Beck Scale for Suicide Ideation).

### Randomization

After filling out the baseline questionnaires, the participants will be randomized to the intervention or control condition using a centralized and computer-based algorithm with a hidden sequence. The algorithm is not accessible or known to the researchers directly involved in the project. The randomization is stratified by sex and level of suicidal ideation (using a cutoff score of ≥16 points on the Beck Scale for Suicide Ideation). A 1:1 allocation ratio will be applied.

### Intervention

Participants assigned the intervention condition will receive a Danish translation of the Dutch intervention, “Living under control” [7].

The intervention and its theoretical background has been described in detail elsewhere [9–11]. It consists of six modules that each takes a week to complete [7]. Every module starts with a theoretical section followed by one compulsory exercise, several optional exercises and a Frequently Asked Questions section. The participants are encouraged to use a minimum of 15 min twice a day to carry out the exercises and they can, through a message system on the website, write to the trial manager if they have any questions or concerns. It is up to each participant if they want to receive any help from the trial manager or just want to go through the intervention without any guidance.

The first of the six modules aims to help the participants to gain more control over their suicidal thoughts. Participants will be encouraged to schedule specific “worry times” during the day, e.g., 8 a.m. and 8 p.m., where they force themselves to think about all the things that upset them. The participants will be asked to perform this exercise every day for the 6 weeks that the intervention lasts. They will also be asked to tally every time they have a suicide-related thought as a means to be more conscious about their thoughts and how often they think about suicide.

In the second module, the participants will develop a crisis plan and practice how to tolerate intense emotions. The third module focuses on how participants can influence their feelings by being aware of negative thoughts. In the fourth module, the participants will be introduced to negative thinking habits and how these might influence thoughts and feelings. In the fifth module, the participants will be offered exercises aimed to help them reformulate negative thoughts. The last module focuses on the future and will help the participant make a relapse prevention plan.

Access to subsequent modules will be available on a weekly basis; e.g., when starting the intervention only the first module is available and the following week the second module will open. The participants in the intervention condition will also have access to the intervention after the first 6 weeks have passed. At the first login to the website, the personal code card, the NemID, is required; after this, participants will be able to choose a username and password. An authorized translation of the intervention into Danish was adapted by an experienced psychologist within the field of suicide prevention and the trial manager.

#### Waiting list

The participants in the control condition will have access to the SOS trial website but not to the modules or the message system. They can access an “Acute Help” page where they can seek help information, and a “My Profile” page where they can read the Informed Consent Form, the participant information and change their login and contact details (these features are also available to the participants in the intervention condition). Once the 32 weeks have passed, the first module of the intervention will be accessible for the participants in the control condition.

All participants can seek help and treatment during the trial. In material and messages with information about the study, the participants are furthermore encouraged to contact The Lifeline or their general practitioner if they are not feeling well.

### Outcomes

#### Primary outcome measure

The primary outcome is a reduction in suicidal thoughts, measured by the Beck Scale for Suicide Ideation. The 19-item self-report scale measures the severity of suicidal thoughts on a scale from 0 to 38 with a higher score indicating higher severity. The Beck Scale for Suicide Ideation is a validated and frequently used measure of suicidal thoughts [12]. As in the Dutch trial, a pre-post difference of 6 points is considered clinically significant [8].

#### Secondary outcome measures

Secondary outcomes cover psychological wellbeing and health care utilization as well as productivity level for paid and unpaid work. In addition, episodes of deliberate self-harm, death by suicide and the participant’s perception and experience of the intervention will be measured.

Level of suicidal thoughts will be measured using the Suicidal Ideation Attributes Scale; a newly developed scale for measuring suicidal thoughts consisting of five questions [7]. We will test the validity of the Danish version of the scale.

Depressive symptoms will be measured using the Major Depression Inventory and the six-item Hamilton Depression Rating Scale. The Major Depression Inventory is developed by the World Health Organization’s Collaborating Center in Mental Health and will be used to generate an *International Classification of Diseases*, *10th edition* (ICD-10) or a *Diagnostic and Statistical Manual of Mental Disorders*, *version 4* (DSM-IV) diagnosis of clinical depression based on the baseline measurement [13, 14]. The six-item Hamilton Depression Rating Scale is an abbreviated version of the original 17-item scale containing six core symptoms of depression [15]. It will be used to assess the severity of a depression [16]. Hopelessness will be measured using an established standard tool, the Beck Hopelessness Scale [17]. The Penn State Worry Questionnaire Past Week will be used to determine the participants’ level of worrying [18]. Quality of life will be measured with the WHO-5 Well-Being Index, which is a well-established and validated questionnaire [19].

Health care utilization registered in the Danish registers will be assessed. Every person in Denmark has a personal identifier that is listed in the Danish registers [20]. Health care utilization at somatic hospitals, outpatient clinics and emergency rooms will be collected from the National Patient Register. Data on contacts to the participant’s general practitioner will, together with services from psychologists or psychiatrists who are subsidized or partly subsidized by the authorities, be obtained from the National Health Service Register. Data on prescribed medication will be obtained from the National Prescription Registry.

Level of paid and unpaid work will be assessed using the Trimbos/IMTA questionnaire for Costs associated with Psychiatric Illness [21].

Demographic data regarding age, sex, education level and relationship status will be collected through the Trimbos/IMTA questionnaire for Costs associated with Psychiatric Illness.

Information on deliberate self-harm episodes will be calculated using a self-report questionnaire and hospital records obtained from the Danish National Patient Register and the Psychiatric Central Research Register.

The participant will, after the intervention, be asked if they have experienced any negative effects related to the intervention and will be encouraged to give feedback; for instance, if they have not completed all six modules they will be asked why. Lastly, the participants will also be given the Internet Evaluation and Utility Questionnaire consisting of 15 questions regarding the usefulness, credibility and helpfulness of the intervention [22].

**Fig. 2:**
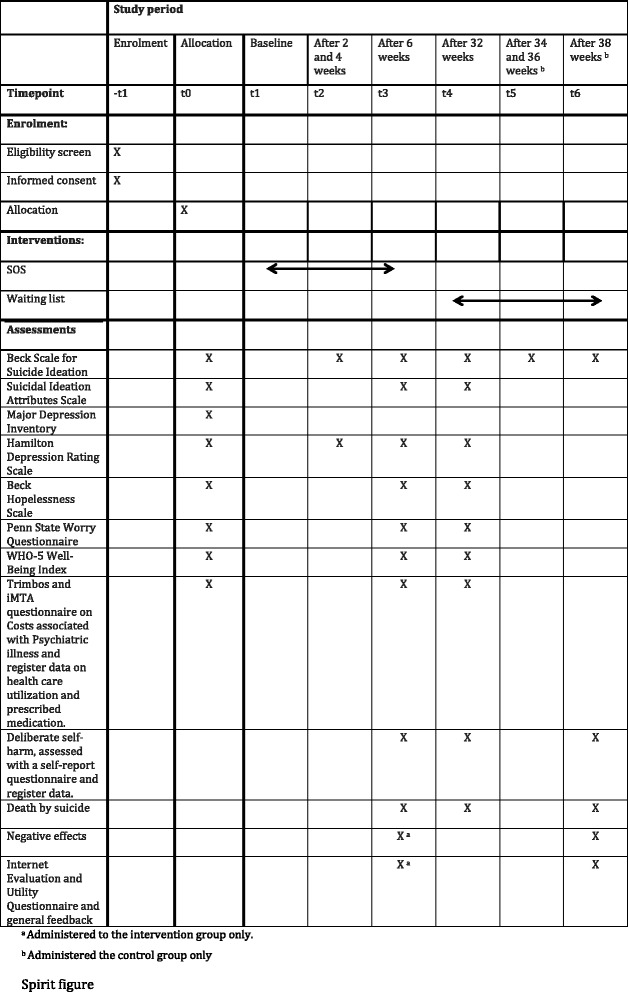
Standard Protocol Items: Recommendations for Interventional Trials (SPIRIT) figure

Data will be collected on how many times the participants log in to the website, how much time they spend on the intervention, how many modules they start and how many messages they send to the trial manager. The self-report questionnaires will be answered on the website. The SPIRIT figure (see Fig. 2) provides an overview of the measures used in the trial and their time points. See the Additional file 1 for the SPIRIT checklist.

### Adverse events

Information on deaths by suicide or other causes of death will be acquired from the Cause of Death Register.

### Statistical methods

The null-hypothesis assumption is that there is no difference between the intervention and the control condition with respect to suicidal thoughts and the secondary outcome measures. Linear mixed models with repeated measurements and an unstructured covariance matrix, will use measurements at baseline, 2, 4 and 6 weeks (post test) with the purpose of modeling changes over time in suicidal thoughts. Mixed models will also be used to assess the long-term effect at 6 months after post test as well as the effect on the participant’s level of depression. This analytical approach handles missing data appropriately using maximum likelihood, under the assumption that data are not missing-not-at-random. The analytical models will include baseline values and stratification variables. Changes in other secondary outcomes will be measured using ANCOVA with multiple imputations. The imputation models will be linear regression for scale variables and binary logistic regression for dichotomous variables. Imputation models will be constructed separately for each randomization group, and include baseline values of the scales in question, stratification variables, variables predicting missing data, and potential auxiliary variables identified for each outcome. Analytical models will include the stratification variables. Robustness of analyses using multiple imputations will be tested using sensitivity analyses instead, replacing missing data with either very low or very high scores for scale variables, and all 0s or all 1s for dichotomous data. Since no missing data are present for register data, imputations will not be necessary for these analyses. Cohen’s *d* test will be used to determine the difference between the intervention and the control condition. In addition, incidence rates of deliberate self-harm will be calculated using a self-report questionnaire and hospital records. A cost-effectiveness analysis will be conducted based on the health care utilization and productivity levels of the observed participants. Lastly, the participant’s evaluation, usage and experience of negative effects during the intervention will be calculated and described.

### Sample size

The sample size was estimated based on the expected effect on suicidal thoughts at 6 weeks. In the Dutch trial, the pooled standard deviation at post test was 0.9 and an effect size of 0.28 (Cohen’s *d*) was observed. If the true difference in mean scores of the intervention and the control condition is 0.30 in the Danish trial, 175 participants in each condition are needed to reject the null hypothesis that there is no difference between the intervention and the control condition at post test on suicidal thoughts with probability (power) 0.8. The type I error probability associated with this test of the null hypothesis is 0.05.

Approximately 90% of participants in the Dutch trial completed all post-test questionnaires. Accounting for a dropout rate of 20%, a total of 438 participants will be needed.

### Blinding

Due to the nature of the intervention, the participants cannot be blinded to the allocation.

During the first 32 weeks of the study period, only participants assigned to the intervention condition will have access to the modules. Thus, the trial manager will not be blinded when receiving messages or phone calls with questions regarding the intervention. Each participant will have a unique Drupal ID number that is not known to the trial manager through the intervention period. When analyzing the data, the trial manager cannot see the participant’s names, but instead only their ID number. The trial manager will thus be blinded when analyzing the study. Only the main developer in the project will have access to all the information.

### Strategies to improve adherence

Adherence in Internet-based therapies is often low; only 21% of the participants completed all six modules in the Dutch trial [7, 23, 24]. Three strategies will be applied to improve adherence: first, the trial manager will inform the participants over the telephone what it implies to participate in the study before they sign the Informed Consent Form. Second, the participants are encouraged to send messages to the trial manager if they have any questions. Third, participants will, through text messages, receive reminders with information when a new module is open and when they need to answer a new set of self-report questionnaires.

In addition, a successful administration of the self-report questionnaires at 6 weeks and 32 weeks will be rewarded with four cinema tickets. The participants assigned to the control condition will, in addition, receive a cinema ticket when they, after 32 weeks of waiting-list status, commence the intervention and answer the post-intervention questionnaires.

### Safety of subjects

Specific measures are taken to secure the safety of subjects during the study period. Firstly, participants will only be enrolled in the study after they provide their telephone number and the number of a contact person (e.g., a family member, a physician or a friend). If the trial manager suspects that a contact person’s telephone number is not valid, she will call the number to check. Secondly, the Beck Scale for Suicide Ideation will be administered every second week over the first 6 weeks to survey the severity of suicidal thoughts. If a participant in either the intervention or the control condition scores ≥27 on the scale, they will receive a telephone call from the trial manager. If the participant cannot be reached during three attempts over 3 days, the participant’s contact person will be contacted. This procedure is communicated to the participant during the enrollment phase. The same procedure will be carried out if a participant ceases to follow the intervention without notifying the trial manager by e-mail or telephone. Thirdly, the SOS trial website lists contact information to psychiatric hospitals and suicide preventive clinics in the event of a crisis. Fourthly, participants will via messages be encouraged to contact The Lifeline or their general practitioner if they are feeling unwell. Fifthly, participants can contact the trial manager by e-mail or telephone if they have questions or concerns. Additionally, a safety protocol has been developed for the study for cases of acute crisis where the trial manager is worried that a participant is in the process of planning a suicidal act. If this happens, the trial manager will, through the website, obtain access to the participant’s personal identifier number, and call the Danish alarm central. They can, with the participant’s personal identifier number and telephone number, track the person and request an ambulance or the police.

## Discussion

This paper describes the study protocol of a randomized controlled trial comparing an Internet-based self-help intervention’s ability to reduce suicidal thoughts with a waiting-list control condition.

Although Denmark has specialized suicide prevention clinics, a relatively stable suicide rate indicates that not all people with suicidal thoughts seek or receive adequate help. It is important to generate evidence on the effectiveness of new interventions with the purpose of implementing effective suicide prevention. In this study, a diagnosis of a major depressive disorder, schizophrenia, personality disorder or substance abuse is not a reason for exclusion. In comparison to the Dutch trial, we have a post-intervention follow-up period and include people with severe suicidal thoughts. The findings obtained will, thus, be generalizable to a wide population segment and will help to generate additional evidence for the effectiveness of Internet-based self-help interventions.

A limitation is that, due to ethical concerns, it is compulsory for participants to use their personal code card; this will compromise anonymity. It is possible that a subset of users, otherwise interested in the intervention, will not be willing to login with their personal code card. Consequently, our findings might not be fully representative of the group that would potentially use the self-help therapy.

Should the online self-help intervention be found to have a non-negative effect, it will be made freely available for all.

### Trial status

Recruitment starts in August 2016 and is anticipated to finish in 2017. The estimated completion date for the final participants is in April 2018.

## Additional file

Additional file 1: SPIRIT Checklist. (DOC 121 kb)

## Abbreviation

SOS trial: The Self-help Online against Suicidal thoughts (SOS) trial

## References

[1] WHO. http://www.who.int/mediacentre/factsheets/fs398/en/. World Health Organization 2016. [cited 2017 Jan 24].

[2] Nock MK, Borges G, Bromet EJ, Alonso J, Angermeyer M, Beautrais A (2008). Cross-national prevalence and risk factors for suicidal ideation, plans and attempts. Br J Psychiatry.

[3] Erlangsen A, Lind BD, Stuart EA, Qin P, Stenager E, Larsen KJ (2015). Short-term and long-term effects of psychosocial therapy for people after deliberate self-harm: a register-based, nationwide multicentre study using propensity score matching. Lancet Psychiatry Elsevier.

[4] Wissing L. “I actually had no wish to die – I just could not figure out life” – The Lifeline –suicide prevention through 20 years. [“Egentlig ønskede jeg ikke at dø – jeg kunne bare ikke finde ud af livet” – Livslinien – selvmordsforebyggelse i 20 år]. Informations Forlag; 2015. ISBN: 9788775144853.

[5] Andrews G, Cuijpers P, Craske MG, McEvoy P, Titov N (2010). Computer therapy for the anxiety and depressive disorders is effective, acceptable and practical health care: a meta-analysis. PLoS One.

[6] Hedman E, Ljótsson B, Lindefors N (2012). Cognitive behavior therapy via the Internet: a systematic review of applications, clinical efficacy and cost-effectiveness. Expert Rev Pharmacoecon Outcomes Res.

[7] van Spijker BAJ, van Straten A, Kerkhof AJFM (2014). Effectiveness of online self-help for suicidal thoughts: results of a randomised controlled trial. PLoS One Public Libr Sci.

[8] Van Spijker BAJ, Cristina Majo M, Smit F, Van Straten A, Kerkhof AJFM (2012). Reducing suicidal ideation: cost-effectiveness analysis of a randomized controlled trial of unguided web-based self-help. J Med Internet Res.

[9] Kerkhof AJFM, van Spijker BAJ, Mokkenstorm JK (2013). Reducing the burden of suicidal thoughts through online cognitive behavioural therapy self help. Suicide prevention and new technologies.

[10] Kerkhof AJFM, Van Spijker BAJ, O’Connor RC, Platt S, Gordon J (2011). Worrying and rumination as proximal risk factors for suicidal behaviour. International handbook of suicide prevention: research, policy and practice.

[11] van Spijker BAJ, van Straten A, Kerkhof AJFM (2010). The effectiveness of a web-based self-help intervention to reduce suicidal thoughts: a randomized controlled trial. Trials.

[12] Beck AT, Steer RA, Ranieri WF (1988). Scale for suicide ideation: psychometric properties of a self-report version. J Clin Psychol.

[13] Bech P, Rasmussen N-A, Olsen LR, Noerholm V, Abildgaard W (2001). The sensitivity and specificity of the Major Depression Inventory, using the Present State Examination as the index of diagnostic validity. J Affect Disord.

[14] Olsen LR, Jensen DV, Noerholm V, Martiny K, Bech P (2003). The internal and external validity of the Major Depression Inventory in measuring severity of depressive states. Psychol Med.

[15] Bech P, Licht RW, Stage KB, Bech-Andersen G, Søndergaard S, Martiny K. Rating scales for affective disorders: compendium [Rating scales for affektive lidelser: kompendium]. Psykiatrisk Forskningsenhed, Psykiatrisk Hospital. 2005. Available from http://docplayer.dk/2479528-Rating-scales-for-affektive-lidelser.html. Accessed 29 Mar 2016.

[16] O’Sullivan RL, Fava M, Agustin C, Baer L, Rosenbaum JF (1997). Sensitivity of the six-item Hamilton Depression Rating Scale. Acta Psychiatr Scand.

[17] Velting DM (1999). Personality and negative expectancies: trait structure of the Beck Hopelessness Scale. Personal Individ Differ.

[18] Stöber J, Bittencourt J (1998). Weekly assessment of worry: an adaptation of the Penn State Worry Questionnaire for monitoring changes during treatment. Behav Res Ther.

[19] Topp CW, Østergaard SD, Søndergaard S, Bech P (2015). The WHO-5 Well-Being Index: a systematic review of the literature. Psychother Psychosom.

[20] Erlangsen A, Fedyszyn I (2015). Danish nationwide registers for public health and health-related research. Scand J Public Health.

[21] Bouwmans C, De Jong K, Timman R, Zijlstra-Vlasveld M, Van der Feltz-Cornelis C, Tan Swan S (2013). Feasibility, reliability and validity of a questionnaire on healthcare consumption and productivity loss in patients with a psychiatric disorder (TiC-P). BMC Health Serv Res.

[22] Ritterband LM, Ardalan K, Thorndike FP, Magee JC, Saylor DK, Cox DJ (2008). Real world use of an Internet intervention for pediatric encopresis. J Med Internet Res.

[23] Karyotaki E, Kleiboer A, Smit F, Turner DT, Pastor AM, Andersson G (2015). Predictors of treatment dropout in self-guided web-based interventions for depression: an “individual patient data” meta-analysis. Psychol Med.

[24] Christensen H, Griffiths KM, Farrer L (2009). Adherence in Internet interventions for anxiety and depression. J Med Internet Res.

